# Assortative mating for human height: A meta‐analysis

**DOI:** 10.1002/ajhb.22917

**Published:** 2016-09-17

**Authors:** Gert Stulp, Mirre J.P. Simons, Sara Grasman, Thomas V. Pollet

**Affiliations:** ^1^ Department of Sociology, University of Groningen / Inter-university Center for Social Science Theory and Methodology (ICS), GroningenThe Netherlands; ^2^ Department of Population HealthLondon School of Hygiene and Tropical MedicineLondonUnited Kingdom; ^3^ Department of Animal and Plant SciencesUniversity of SheffieldSheffieldUnited Kingdom; ^4^ Department of Experimental and Applied PsychologyVU University AmsterdamAmsterdamThe Netherlands

**Keywords:** stature, body size, assortative mating, mate choice, meta‐analysis

## Abstract

**Objectives:**

The study of assortative mating for height has a rich history in human biology. Although the positive correlation between the stature of spouses has often been noted in western populations, recent papers suggest that mating patterns for stature are not universal. The objective of this paper was to review the published evidence to examine the strength of and universality in assortative mating for height.

**Methods:**

We conducted an extensive literature review and meta‐analysis. We started with published reviews but also searched through secondary databases. Our search led to 154 correlations of height between partners. We classified the populations as western and non‐western based on geography. These correlations were then analyzed via meta‐analytic techniques.

**Results:**

148 of the correlations for partner heights were positive and the overall analysis indicates moderate positive assortative mating (*r* = .23). Although assortative mating was slightly stronger in countries that can be described as western compared to non‐western, this difference was not statistically significant. We found no evidence for a change in assortative mating for height over time. There was substantial residual heterogeneity in effect sizes and this heterogeneity was most pronounced in western countries.

**Conclusions:**

Positive assortative mating for height exists in human populations, but is modest in magnitude suggesting that height is not a major factor in mate choice. Future research is necessary to understand the underlying causes of the large amount of heterogeneity observed in the degree of assortative mating across human populations, which may stem from a combination of methodological and ecological differences.

## 1 Introduction

Francis Galton concluded in 1886, that “men and women of contrasted heights, short and tall or tall and short, married just about as frequently as men and women of similar height, both tall or both short” and that stature is “little entangled with … marriage selection” (Galton, 1886, p. 251), thus suggesting that there is no assortative mating for stature. This conclusion that mates do not resemble one another in terms of their heights may have been premature, however, because of the “possibility of the records of height having been frequently drawn up in a careless fashion,” which according to Pearson in his biography on Galton, may be due to “amateur measuring of stature in women, when high heels and superincumbent chignons were in vogue” (Pearson, 1930, p. 18). Subsequent analyses by Pearson (1930) suggested assortative mating in this sample, and many more recent studies have observed such non‐random mating with respect to stature (e.g., review in Spuhler, 1982). Galton's work on height and heredity laid the foundation for future statistical concepts, but even today the question remains whether assortative mating for stature occurs in all human populations, and if so, to what extent?

Why would there be non‐random patterns of height in human couples? Mate choice is likely to play an important role, as a plethora of preference‐studies have shown that height matters, when rating potential partners for attractiveness (see Courtiol, Raymond, Godelle, & Ferdy, 2010; Stulp & Barrett, 2016, for reviews). Such studies reveal a clear assortative preference: taller men and women prefer taller partners than do shorter men and women. Other preference rules for height do exist (Courtiol et al., 2010; Fink, Neave, Brewer, & Pawlowski, 2007; Pawlowski, 2003; Stulp, Buunk, Kurzban, & Verhulst, 2013a; Stulp, Buunk, Pollet, 2013b; Stulp, Buunk, Pollet, Nettle, & Verhulst, 2013c), but these are not incompatible with assortative preferences and can also lead to assortative mating. Indeed, in a speed‐dating study, verbalized preferences for height combined with mutual mate choice revealed how such preferences can lead to assortative pairing (Stulp et al., 2013a).

Assortative mating can have important consequences for the direction and strength of natural selection on traits (Jiang, Bolnick, & Kirkpatrick, 2013; Kirkpatrick, 2000). Assortative mating increases trait variance in a population when the trait is heritable, because offspring born to different parents show more trait divergence under assortative compared to random mating. A trait subject to assortative mating can thus increase the response to directional and disruptive natural selection (Fox, 2003; van Doorn, Edelaar, & Weissing, 2009). Conversely, it can potentially disrupt balancing selection and reduce migration load contributing to speciation (Fox, 2003; Lenormand, 2002). Assortative mating can therefore also aggravate intralocus sexual conflict pushing trait values to extremes where sexual conflict is highest. It could therefore contribute to the maintenance of unresolved sexual conflicts, a phenomenon that continues to puzzle evolutionary biologists (e.g., Fox, 2003). Intralocus sexual conflict over human height is present (at least phenotypically: Stearns, Govindaraju, Ewbank, & Byars, 2012; Stulp, Kuijper, Buunk, Pollet, & Verhulst, 2012), making the investigation into assortative mating for this trait particularly interesting.

We conduct a meta‐analysis on 154 effect sizes from 43 different countries to test for assortative mating for human height and quantify its strength. Some studies suggest that we should not expect assortative mating to be universal (e.g., Sear & Marlowe, 2009; Sorokowski & Butovskaya, 2012; Sorokowski & Sorokowska, 2012; Sorokowski, Sorokowska, Butovskaya, Stulp, Huanca, & Fink, 2015; Sorokowski, Sorokowska, Fink, & Mberira, 2011), and may be restricted to western populations. We therefore test whether effect sizes are higher in western societies.

## 2 Methods

### 2.1 Literature search

We searched through Pubmed, PMC, and Web of Science with the search terms “assortative mating height”, “husband‐wife correlations stature”, “assortative pairing human”, “height assortment”, “stature assortment”,” couple stature human”, “phenotypic matching human”, “family resemblance height”, and “family resemblance stature.” For Pubmed and PMC, we used the RISmed package in R (Kovalchik, 2015), to extract the records (309 unique records, 15 February 2016). For Web of Science we used the advanced search tool (using the Boolean AND operator between the search words) (365 records, 16 February 2016). All records were then assessed for relevance based on the title and abstract. If deemed relevant, we examined the full paper where possible and included those records reporting a correlation coefficient for assortative mating for height. In addition, we examined previous reviews on assortative mating for height (Spuhler, 1968; Susanne & Lepage, 1988; Wolański, 1994). See Supporting Information for a list of studies that were obtained through searching these databases, as well as the list of studies that were included in our database. Given the breadth of the field, spanning from human biology and genetics to demography, sociology, and psychology, we did not publish a call for unpublished papers. This decision was made prior to analyzing the data gathered.

SG and TVP extracted data on the study population, correlation coefficient, and added notes pertaining to statistics (e.g., whether the association was corrected for age or not). The only inclusion criteria were that the study reported on a correlation coefficient for stature (body height) between (human) partners. We did not code or differentiate between studies using measured vs. self‐reported height, as we assume that these are highly correlated (Spencer, Appleby, Davey, & Key, 2002). In addition, several studies do not clearly report how height was measured. Studies on height ratios (e.g. standing to sitting ratio; Hasstedt, 1995) were excluded. We were unable to derive effect sizes from a record reporting on a twin sample (Hirschhorn et al., 2001) and were unable to locate a potentially relevant paper from our literature search (Bergman & Koniarek, 1999). The following studies (including reviews that reported on several studies) were included in the meta‐analysis: Pearson and Lee, 1903; Susanne, 1967, 1977, 1979; Spuhler, 1968, 1982; Johnston, 1970; Pollitzer et al., 1970; Baldwin and Damon, 1973; Crognier, 1973; Harrison, Gibson, and Hiorns, 1976; Hill, Rubin, and Peplau, 1976; Mueller and Malina, 1976; Roberts, Billewicz, and McGregor, 1978; Garn, Cole, and Bailey, 1979; Chrzastek‐Spruch, 1979; Nance, Corey, and Eaves, 1980; Price and Vandenberg, 1980; Kaur and Singh, 1981; Pieper, 1981; Malina, Selby, Buschang, Aronson, and Little, 1983; Annest, Sing, Biron, & Mongeau, 1983; McManus and Mascie‐Taylor, 1984; Pennock‐Román, 1984; Sharma and Sharma, 1984; Ahmad, Gilbert, & Naqui, 1985; Province and Rao, 1985; Staessen et al., 1985; Byard, Poosha, and Satyanarayana, 1985; Byard, Mukherjee, Bhattacharya, Russell, and Rao, 1989; Hutchinson and Byard, 1987; Mascie‐Taylor, 1987; Nagoshi and Johnson, 1987; Okada, 1988; Stark, Salzano, and DaRocha, 1990; Wolański et al., 1990; Tambs et al., 1992; Sutton, 1993; Sanchez‐Andres and Mesa, 1994; Wolański, 1994; Dasgupta, Dasgupta, and Daschaudhuri, 1997; Ginsburg, Livshits, Yakovenko, and Kobyliansky, 1998; Luo, Albertsson‐Wikland, and Karlberg, 1998; To, Cheung, and Kwok, 1998; Eaves et al., 1999; Price, Reed, and Guido, 2000; Dalmia and Lawrence, 2001; Eckman, Williams, Nagoshi, 2002; Al‐Kandari, Crews, & Poirier, 2002; Xu et al., 2002; Hur, 2003; Mukhopadhyay et al., 2003; Raychaudhuri, Ghosh, Vasulu, and Bharati, 2003; Silventoinen, Kaprio, Lahelma, Viken, and Rose, 2003; Salces, Rebato, and Susanne, 2004; Heude et al., 2005; Knuiman, Divitini, and Bartholomew, 2005; Ellis et al., 2007; Godoy et al., 2008; Sear and Marlowe, 2009; Ajala et al., 2011; Zietsch, Verweij, Heath, and Martin, 2011; Becker, Touraille, Froment, Heyer, and Courtiol, 2012; Seki, Ihara, and Aoki, 2012; Keller et al., 2013; Stulp et al., 2013b, 2013c; Stulp, Mills, Pollet, and Barrett, 2014; Uchida, Matsuo, Hori, Hasegawa, and Takahashi, 2013; Ponzo and Scoppa, 2015; Prichard et al., 2015; and Tenesa, Rawlik, Navarro, and Canela‐Xandri, 2016.

In cases where both the unadjusted and age‐adjusted Pearson correlations were available (e.g., Malina et al., 1983) only the unadjusted correlation was used to allow studies to be compared quantitatively. For two studies (Ginsburg et al., 1998; Pollitzer et al., 1970) only the minimum sample sizes were available and these were used. For one study (Pearson & Lee, 1903), a range of sample sizes was available, of which we took the midpoint. Some studies do not report the sample size of the husband‐wife correlation, but did report sample sizes of their children and the parental height correlates; for these studies, we assumed that the number of children matches the *N* of husband‐wife correlations and that we are dealing with biological parents. Some papers report on the same sample and were therefore excluded (e.g., National Child Development study; Power & Elliott, 2006). For one study (Silventoinen et al., 2003), we calculated the weighted average of the Pearson correlation coefficient. One study on a polygynous sample (Roberts et al., 1978), reported three estimates for assortative mating, we used the estimate over the mean height of the wives. In cases where we had a correlation coefficient but no *N* or *SE* estimate were available, we searched reviews and used those if reported. We contacted the corresponding authors when their contact details were available, trying to obtain complete information for as many cases as possible. When only *r* and *SE* were available and a specific *P*‐value was not reported (*N* = 2), we approximated *N* (*SE*, *N*, and *r* are related to one another in the following approximate way: ${SE}_{r}=\sqrt{\frac{1-r^{2}}{N-2}}$).

For 24 out of 26 studies reported in the review by Wolański (1994), a correlation coefficient was available but not a sample size or standard error. In seven of these cases, a *p* value was reported in the form of a significance category (e.g., *P* < .05), and in these cases we calculated a lower limit sample size by using the formula $r=\frac{t}{\sqrt{t^{2}+df}}$ from Nakagawa and Cuthill (2007) (substituting a *t* of 1.96, 2.58, or 3.59, in the case that respectively *P* < .05, *P* < .01, or *P* < .001 was reported, and substituting *df* for *N*−1). For the remaining 17 cases, for which also no significance category was available, we imputed the median sample size across all nine studies reported by Wolański (1994) on which such information was available or computed (Median *N* = 68). All imputed sample sizes were rounded such that only integers were used. The dependent variable in our meta‐analysis was the Fisher transformed correlation coefficient *Z_r_*, the distribution of which follows a normal distribution ( $Z_{r}=0.5\;ln\left[\frac{1+r}{1-r}\right]$, with ${SE}_{Zr}=\frac{1}{\sqrt{N-3}}$; Nakagawa & Cuthill, 2007).

**Figure 1:**
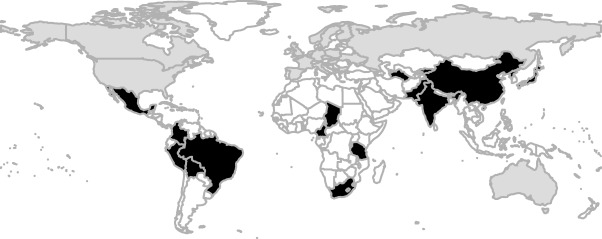
Samples were drawn from countries shaded in black (classified as non‐Western; *N* = 23) and grey (classified as Western; *N* = 20)

We used the UN region to code “populations” as ‘western' based on geographical location (Europe (code 150), Northern America (code 021), Australia and New Zealand (code 053)) (http://unstats.un.org/unsd/methods/m49/m49regin.htm), this corresponds to the UN classification as “developed region”, with the exception of Japan (code 392) (which is classified as “developed,” but is characterized as “non‐western” here). Note that the codes may not be an accurate reflection of either western of non‐western, since these are based on *current* geographical codes. While some countries can be considered as (culturally) western nowadays, this does not necessarily imply they have been so in the past. Also, note that a subpopulation within a ‘western’ population could be wrongfully considered ‘western’ (e.g., Native Americans in the US). Additionally, note that geopolitical boundaries have changed between 1899 and 2016 (e.g., former USSR countries) and that we refer to *current* geocodes. Nonetheless, we feel that the UN region‐coding scheme at least provides an unambiguous, replicable differentiation of populations by region and results can be interpreted accordingly. See Figure 1 for a world map with all sampled populations.

Publication year is used to examine trends over time, assuming close correspondence to when the data were collected. This remains a proxy, but actual sampling times were unfortunately not available for all of the studies. Effect sizes tend to decline over time if publication bias is present, but in this case, a lower degree of assortative mating might also be expected in older studies further away from modernizing influences that might contribute to assortative mating in current western society.

### 2.2 Analysis

The collected correlation coefficients were subjected to a mixed‐effects meta‐analysis, with Fishers *Zr* as dependent variable (Nakagawa & Cuthill, 2007; which was back‐transformed to *r* for presentation purposes). We employed mixed‐effects meta‐analysis using the *metafor* package (Viechtbauer, 2010) in R including random effects for author(s) (91 levels) and country in which the study was performed (43 levels) to correct for pseudoreplication (see the Supporting Information for the dataset used for analyses and Figures). We included the inverse variance weights based on sample size (*N*−3). Fixed moderators included were publication year (mean centered) and whether the study was performed in a western population or not, and we present the estimates from this full model. The interaction between publication year and whether a study was from a western population or not contributed very little to the model (estimate of slope difference in western populations −.0001± .0008, *P* = .89), and was not included in the final model.

False convergence was not detected for any of the models based on the likelihood surface profiles. Publication bias was evaluated using a rank test and funnel plot (see Supporting Information Figure S1), and these did not indicate any such bias (*Kendall's tau*=–.047, *P* = .39).

## 3 Results

Out of 154 within‐pair correlations for height, 148 were positive and only six were negative. These six samples were from Turkmenia, Native American populations (Seminole, Navaho), the Solomon islands (Kwaio, Lau) and Rural Western Bengal (all samples: *N* < 120). Not surprisingly then, across all‐studies significant moderate assortative mating for height (*r* = .23, *95%CI*: .21–.26, *P* < .0001) was observed based on the model without any moderators.

**Figure 2:**
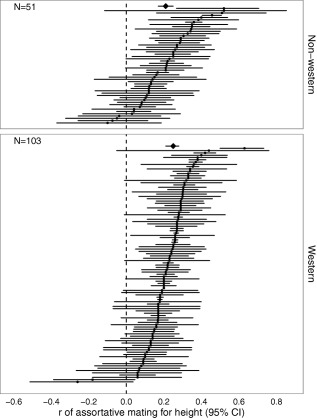
Degree of assortative mating (*r*) in Western and non‐Western populations. Circles are individual populations (although multiple estimates per study are possible), and diamonds are estimated overall effect sizes from the meta‐analysis (bars reflect 95% confidence intervals)

In both western (*r* = .25, *95%CI*: .21–.28, *P* < .0001) and non‐western cultures (*r* = .21, *95%CI*: .17–.25, *P* < .0001) assortative mating was observed (see Figure 2). Although assortative mating seemed somewhat stronger in western compared to non‐western populations, this did not reach statistical significance (estimate (±*SE*): .038 ± .024, *P* = .12). The timing of publication had no effect on the degree of assortative mating (.00003 ± .0004, *P* = .95).

In meta‐analyses, heterogeneity is the deviation from normal sampling variance as estimated through meta‐analysis and provides a quantitative insight in whether there is variance in the effect sizes within a meta‐analysis that could be explained by unknown moderators or whether the observed variance is mostly due to sampling error (Nakagawa & Santos, 2012; for example, with a lower number of studies included, there will be higher variance in effect sizes). Considerable residual heterogeneity was observed in the overall model (*I^2^*= 93%, *Q*(151)=918, *P* < .0001), suggesting considerable scope for unknown moderating variables explaining variation between studies in either methodology or because of cultural and biological factors. Interestingly, heterogeneity was substantially smaller in non‐western populations (I^2^ = 76%, *Q*(50)=224, *P* < .0001) compared to western populations (I^2^ = 92%, *Q*(102)=699, *P* < .0001).

## 4 Discussion

Mates tend to resemble one another in a variety of traits (see e.g., Jiang et al., 2013 for review), and also in humans such positive assortative mating has been widely described for many traits, including age, religiosity, personality, and weight (e.g., Zietsch et al., 2011). Here we show on the basis of 154 correlations, and in contrast to Galton's conclusion that stature is “little entangled with … marriage selection “(Galton, 1886, p. 251), that there was a moderate amount of assortative pairing for height across human populations (*r* = .23). The strength of this assortment appears to be relatively constant over time.

Mate choice is an obvious candidate for the observed assortative mating, since a plethora of studies suggest that taller individuals prefer taller partners (see Courtiol et al., 2010 and Stulp & Barrett, 2016 for reviews). Furthermore, assortative pairing with respect to height has shown to arise out of mutual mate choices during speed‐dating (Stulp et al., 2013a). The observation that the magnitude of assortative mating is small (although very similar to those observed in animals with respect to body size; Jiang et al., 2013), suggests that height is not an important factor in mate choice, and/or that many other factors play a role. This is also very much in line with mate choice studies on the role of stature: while height was a factor in the popularity of speed‐daters, it was not one of great importance, and many individuals were chosen as dates even if their height fell outside the range preferred by the chooser (Stulp et al., 2013a). Nonetheless, preferences for height resulted in assortment for height between dates, giving support to the role of mate choice in the non‐random mating patterns related to stature.

Assortative mating need not be a consequence of assortative preferences for height. A previous simulation study showed, for example, that simply a male‐taller norm (e.g., as a woman, only accept men who are taller than yourself as a partner) would result in assortative mating, without the couples explicitly pairing on similar (relative) height (Stulp et al., 2013c). Interestingly, the degree of assortative mating in such a case (i.e., in a situation where all couples abide by the male‐taller norm) is much stronger than observed here, suggesting yet again, that height, or even the male‐taller norm, is not particularly important when considering a partner.

The importance of the role of stature in mate choice might also explain the observation that the degree of assortment was slightly stronger (albeit not significantly) in western (*r* = .25) compared to non‐western populations (*r* = .21), although significant positive assortative mating was observed in both. Preferences for stature in non‐western populations have been shown to be much less consistent compared to western populations, and sometimes even non‐existent (e.g., Sear & Marlowe, 2009; Sorokowski & Butovskaya, 2012; Sorokowski & Sorokowska, 2012; Sorokowski et al., 2015, 2011). Less pronounced assortative mating may well be a consequence of the lower value placed on height as a partner characteristic. However, given that the strength of assortative mating was not statistically different in western compared to non‐western populations was statistically indistinguishable, there is also the possibility that the lack of assortative mating observed in the latter populations has been a consequence of typically low sample size per study, compared to those from western populations.

More generally, population‐differences in the value of height in mate choice may explain the large variation in assortative mating that is observed across studies. Indeed, much of the variability in assortative mating remains unexplained (when expected sampling variance is accounted for), in particular in western populations that are supposedly more homogenous. Future research is necessary to understand the underlying causes of this variability (Stulp & Barrett, 2016), which may stem from a combination of measurement differences (e.g., measured versus self‐reported height), samples (e.g., twin designs vs. other), and as of yet unknown cultural or ecological differences. Future research could also examine non‐linear patterns in height, as there is some evidence for the idea that the degree of assortative mating is different across the height continuum (e.g., McManus & Mascie‐Taylor, 1984; Stulp et al., 2014). Such non‐linear patterns will inevitable decrease the strength of the assortative mating as measured by a correlation coefficient. Thus, when such non‐linear patterns are strong and a low correlation coefficient is observed, this may lead to the erroneous conclusion that assortative mating for stature is not important.

The division between western (collapsing Europe, North America, and Australia in a single category) and non‐western (collapsing Southern American, Asian, and African countries in a single category) is rather crude. In particular, the latter category “non‐western” is rather diverse. The reason for maintaining this particular distinction is two‐fold: (1) previous research has made explicit claims about how western populations may vary from non‐western ones (e.g., Sear & Marlowe, 2009; Sorokowski & Butovskaya, 2012; Sorokowski & Sorokowska, 2012; Sorokowski et al., 2015, 2011); (2) the number of non‐western populations from different parts of the world (see Figure 1) are too limited to make further useful classifications, nor are there specific a priori hypotheses to make such a classification. As an example, for the entire continent of Africa, there were only eight studies from five different countries. It is clear that when more estimates of assortative mating become available, in addition to characteristics of the sampled populations, more fine‐grained analyses can be performed that might be able to explain some of the heterogeneity in results.

Although we believe mate choice in humans is an obvious and likely candidate for the assortative mating observed here, it is important to note that partner similarity in height can also arise through different processes (Courtiol et al., 2010). For instance, when height is correlated to traits that *are* involved in assortative mating (e.g., ethnicity, education). We believe this is unlikely to account for the observed assortment in its entirety for several reasons. First, assortative mating for height is relatively unaffected when controlling for husband and wives' education, health, and income (known correlates of height), suggesting that husband‐wife assortment for height is likely a consequence of mate choice for the trait itself (Stulp et al., 2014). Second, a study on a large sample of twins, their partners, and parents, found evidence that assortative mating was most likely due to initial choice (Zietsch et al., 2011). Third, inter‐ethnic imbalances in marriages are well explained by preferences for stature, suggesting that mate choice for height really is a driving factor (Belot & Fidrmuc, 2010). Of course, there may be other, yet unidentified, traits correlated with height, which could also account for spousal similarity in height, without height being directly selected for in mate choice. One particular case may be the location of living: height varies geographically and people mate locally, which may cause assortative mating in stature without any process of mate choice for height involved. Yet, even within local samples, assortative mating for height is observed (e.g., student samples from one particular city; Stulp et al., 2013b), suggesting that geography cannot be the sole explanation (see Stulp et al., 2013c for further discussion).

Regardless of the mechanisms that result in assortative mating for height in humans, its effect on the strength of natural selection is the same. Through positive assortment, the genetic response to selection increases on height itself and genetically correlated traits. Assortative mating is therefore also predicted to aggravate intralocus sexual conflict when the trait is under sexually antagonistic selection. Recent studies show that stature is indeed subject to sexually antagonistic selection: in the US height shows a curvilinear relationship with reproductive success in men and a negative relationship in women (Stearns et al., 2012; Stulp & Barrett, 2016; Stulp et al., 2012). Given such relationships, assortative mating for stature increases the genetic conflict, and, particularly for taller individuals, assortment for height seems suboptimal in terms of offspring fitness. Interestingly, something different seems to hold for the Netherlands, where taller men and average height women tend to have the largest number of children (Stulp et al., 2015), and where linear assortative pairing for shorter individuals may be suboptimal in terms of offspring fitness. Assortative mating for height therefore poses a currently unresolved paradox in the face of intralocus sexual conflict. Other (presently unknown) benefits could maintain assortative mating or it could emerge from the discrepancy between mate preferences of both sexes and actual pair formation (Stulp et al., 2013a). The degree of assortative mating for height and individual selection gradients determine the response to selection. Understanding such relationships may be important for understanding whether and to what degree Darwinian selection on height contributes to (future) variation in height across the globe (Stulp & Barrett, 2016).

## Supporting information

Supporting Information Figure 1

Supporting Information 1

Supporting Information 2
